# Genetic associations between serum low LDL-cholesterol levels and variants in *LDLR*, *APOB*, *PCSK9* and *LDLRAP1* in African populations

**DOI:** 10.1371/journal.pone.0229098

**Published:** 2020-02-21

**Authors:** Mahtaab Hayat, Robyn Kerr, Amy R. Bentley, Charles N. Rotimi, Frederick J. Raal, Michèle Ramsay

**Affiliations:** 1 Sydney Brenner Institute for Molecular Bioscience, Faculty of Health Sciences, University of the Witwatersrand, Johannesburg, South Africa; 2 Division of Human Genetics, National Health Laboratory Service and School of Pathology, Faculty of Health Sciences, University of the Witwatersrand, Johannesburg, South Africa; 3 Center for Research on Genomics and Global Health, National Human Genome Research Institute, National Institutes of Health, Bethesda, Maryland, United States of America; 4 Carbohydrate and Lipid Metabolism Research Unit, Faculty of Health Sciences, University of the Witwatersrand, Johannesburg, South Africa; University of Tampere, FINLAND

## Abstract

Non-communicable diseases, including cardiovascular diseases (CVDs), are increasing in African populations. High serum low density lipoprotein cholesterol (LDL-cholesterol) levels are a known risk factor for CVDs in European populations, but the link remains poorly understood among Africans. This study investigated the associations between serum LDL-cholesterol levels and selected variants in the low density lipoprotein receptor (*LDLR*), apolipoprotein B (*APOB*), proprotein convertase subtilisin/kexin type 9 (*PCSK9*) and low density lipoprotein receptor adaptor protein 1 (*LDLRAP1*) genes in some selected African populations. Nineteen SNPs were selected from publicly available African whole genome sequence data based on functional prediction and allele frequency. SNPs were genotyped in 1000 participants from the AWI-Gen, study selected from the extremes of LDL-cholesterol level distribution (500 with LDL-cholesterol>3.5 mmol/L and 500 with LDL-cholesterol<1.1 mmol/L). The minor alleles at five of the six associated SNPs were significantly associated (P<0.05) with lower LDL-cholesterol levels: *LDLRAP1* rs12071264 (OR 0.56, 95% CI: 0.39–0.75, P = 2.73x10^-4^) and rs35910270 (OR 0.78, 95% CI: 0.64–0.94, P = 0.008); *APOB* rs6752026 (OR 0. 55, 95% CI: 0.41–0.72, P = 2.82x10^-5^); *LDLR*: rs72568855 (OR 0.47, 95% CI: 0.27–0.82, P = 0.008); and *PCSK9* rs45613943 (OR = 0.72, 95% CI: 0.58–0.88, P = 0.001). The minor allele of the sixth variant was associated with higher LDL-cholesterol levels: *APOB* rs679899 (OR 1.41, 95% CI: 1.06–1.86, P = 0.016). A replication analysis in the Africa America Diabetes Mellitus (AADM) study found the *PCSK9* variant to be significantly associated with low LDL-cholesterol levels (Beta = -0.10). Since Africans generally have lower LDL-cholesterol levels, these LDL-cholesterol associated variants may be involved in adaptation due to unique gene-environment interactions. In conclusion, using a limited number of potentially functional variants in four genes, we identified significant associations with lower LDL-cholesterol levels in sub-Saharan Africans.

## Introduction

The incidence of non-communicable diseases (NCDs) is increasing in Africa [1] and in 2016, 34% of all deaths in Africa were attributed to NCDs [2]. The most prevalent group of NCDs is cardiovascular disease (CVD) for which the common risk factors include dyslipidaemia, diabetes, hypertension and obesity, in combination with smoking, excessive alcohol use and low socioeconomic status [3–5]. High levels of LDL-cholesterol have also been known to be a major contributing factor to the development of CVD.

Investigating genetic associations with LDL-cholesterol levels among sub-Saharan Africans is the focus of this study. LDL-cholesterol levels have a multifactorial aetiology, where environmental factors and variants in many different genes contribute to determining levels of LDL-cholesterol [6,7]. We aimed at selecting variants potentially associated with LDL-cholesterol levels. We did this by identifying common functional variants in genes associated with the monogenic form of high LDL-cholesterol levels–familial hypercholesterolaemia (FH). FH, an autosomal dominant trait, is one of the most common single gene disorders, with a worldwide prevalence of about 1 in 200–250 [8], and is often caused by deleterious variants in *LDLR*, *APOB*, *PCSK9* or *LDLRAP1* [9]. The genetic contribution to LDL-cholesterol levels has not been extensively investigated in African populations and variants in these four genes could potentially influence LDL-cholesterol levels in African populations. Using these genes as potential candidates, we investigated genetic associations with LDL-cholesterol levels as a complex multifactorial trait in African populations. We did this by using an extreme phenotype categorical study design. We compared a group of individuals with high levels of LDL-cholesterol to a group of individuals with low levels of LDL-cholesterol.

Several genome-wide association studies (GWASs) have been performed to identify variants associated with LDL-cholesterol levels [10–17]. However, most of these studies were conducted in European populations with a limited number in African Americans. A meta-analysis of >100,000 European individuals found 22 loci associated with LDL-cholesterol levels including *LDLR*, *APOB*, *PCSK9* and *LDLRAP1* [18]. Studies in African Americans have replicated some of these associations, and in some cases, significantly narrowed down the size of the associated regions, due to generally lower linkage disequilibrium in African populations [19–25]. However, African specific variants that alter LDL-cholesterol levels have not been fully investigated.

Africa is generally a genetic data-scarce region; therefore, large studies on the genetic aetiology of LDL-cholesterol level variation in African populations are few. The genes chosen for this study were based on data from previous associations in European populations and we genotyped selected variants. Ideally, in an African setting, sequence based-approaches are preferable as they have the potential to identify novel African genetic contributions to the variation in lipid levels.

The aim of this study was to investigate whether selected genetic variants in genes previously associated with LDL-cholesterol levels show association in African populations. We identified five variants associated with low LDL-cholesterol levels and one variant associated with high LDL-cholesterol levels. The African American Diabetes Mellitus (AADM) study was used as a replication cohort [26,27]. Given the clinical significance of the high LDL-cholesterol phenotype, this study provides information relevant to an African setting.

## Methods

### Participants

Participants came from six sites in four African countries: Kenya, South Africa, Ghana and Burkina Faso. They were selected from the H3Africa AWI-Gen Collaborative Center (**A**frica, **W**its-**I**NDEPTH Partnership for **GEN**omic studies), an established project investigating genomic and environmental factors that influence cardio-metabolic disease risk in rural and urban Africans [28]. From ~10,000 AWI-Gen participants, 500 participants with high fasting LDL-cholesterol levels (> 3.5 mmol/L) and 500 participants with low LDL-cholesterol levels (< 1.1 mmol/L) were selected to represent the “cases” and “controls”, respectively. The thresholds were set based on the distribution of LDL-cholesterol levels in the AWI-Gen cohort. The rationale was that because all individuals are born with an LDL-cholesterol level of approximately 1.1 mmol/L, [29] a value lower than this suggests a genetic aetiology. A recent meta-analysis on African data used a cut-off of 3.3 mmol/L for high LDL-cholesterol [30], therefore, the high cut-off of > 3.5 mmol/L was appropriate for our study. The age range in our study was between 35 and 80 years. Participants were excluded if they had diabetes, a BMI >35, had problematic alcohol use or were on medication for lipidaemia. The AWI-Gen study was approved by the Human Research Ethics Committee (HREC) (Medical) of the University of the Witwatersrand (Wits), in accordance with the Declaration of Helsinki principles (protocol number M121029), renewed in 2017 (protocol number M170880). This study was approved as an MSc research project by the HREC (Medical) (protocol number M160833).

The data collection for the AWI-Gen study is described by Ali, *et al*., 2018 [31]. Briefly, serum LDL-cholesterol and glucose were analysed with a Randox Daytona Plus Clinical Chemistry analyser (Crumlin, Northern Ireland) using colorimetric assays. The coefficient of variation of the laboratory measurement for lipids and glucose was less than 1.5% and 2.3%, respectively. Body Mass Index (BMI, kg/m^2^) was calculated from height and weight measurements. Classification of diabetes was guided by the standards set by the American Diabetes Association [32]. It was defined as the presence of one or more of the following conditions: previous diagnosis by a health care provider (which excluded gestational diabetes), taking medication for the condition, or a fasting blood glucose level of ≥ 7.0 mmol/L. Alcohol consumption was categorised into: never consumed; current non-problematic consumer; current problematic consumer; former consumer. Problematic drinking was determined according to the CAGE questionnaire [33], where four questions related to potential problematic alcohol consumption were asked, and categorised as problematic if the participant answered “yes” to at least two of them.

### Candidate gene and variant selection

Variants in *LDLR*, *APOB*, *PCSK9* and *LDLRAP1* were identified using publicly available whole genome sequence (WGS) data from African participants in the 1000 Genomes Project (KGP) and the African Genome Variation Project (AGVP). The variants were selected on the basis of *in silico* functional prediction and allele frequency in African populations and genotyped in a group of 1000 AWI-Gen participants, half with low and half with high LDL-cholesterol levels. The variants were tested for association in a case:control study design with high (cases) compared to low (controls) LDL-cholesterol levels, correcting for multiple testing and considering potential confounders.

The four genes are known to be associated with monogenic FH, and in some cases also with multifactorial LDL-cholesterol levels. Variants in these genes were extracted in VCF file format from WGS data of African population samples available from KGP and the AGVP. A region including the genomic sequence of each gene, plus a 1000bp flanking region on either side, was screened for variants. A total of 975 individuals from eight African populations were included in the investigation: 655 WGS from KGP and 320 WGS from AGVP.

A total of 3541 variants were identified. The variants were functionally annotated using CADD [34] and Ensembl’s VEP [35] to identify potentially deleterious variants. Sequences from KGP and AGVP were mapped to GRCh37, therefore VEP was used on Ensembl’s archive site for GRCh37. Variants with a CADD score >10, SIFT score <0.05 or PolyPhen score >0.5 were selected as potentially deleterious.

To increase the power of the association analysis, only variants that were observed in at least six of the eight populations were chosen. Furthermore, the variants were filtered in two stages. Firstly, variants with at least one deleterious score, and being either a missense, start/stop, gain/loss, exonic or regulatory variant, were selected. Secondly, variants with a minor allele frequency (MAF) in African populations (according to dbSNP) of between 10% and 45% were selected to boost the power of the analysis. Linkage disequilibrium (LD) was assessed for the selected variants and no pairs were in strong LD (Haploview, r^2^>0.4) [36].

### Genotyping

The Agena Bioscience MassARRAY genotyping platform was used to genotype 19 selected SNPs. This service was provided by Inqaba Biotech in Pretoria, South Africa. The DNA used for the genotyping was obtained from the Biobank based at the Sydney Brenner Institute for Molecular Bioscience (SBIMB), Johannesburg, South Africa. The DNA concentration for each of the 1000 samples was normalised to ~30 ng/μl and ~10 μl DNA was provided. The MassARRAY system software was used to test whether variants of interest are likely to be successfully genotyped.

### Data analysis

PLINK 1.9 [37] was used. The genotype data was separated into cases (high LDL-cholesterol levels) and controls (low LDL-cholesterol levels) so that logistic regression could be carried out. Quality control was performed and samples with >17/19 missing SNP data were excluded from further analysis. SNP variants with >104/998 (>10%) missingness, Hardy-Weinberg equilibrium (HWE) P<0.005, differential missingness <1x10^-5^ and MAF <0.01 were excluded from further analysis. Quality control measures were derived from Marees *et al*., (2018) [38] with slight modifications to fit this small dataset.

### Association analysis

We used logistic regression analysis using 14 SNPs in the four genes of interest. Associations were corrected for multiple testing using the Benjamini-Hochberg method. All associations with P<0.05 after adjustment were considered significant. The odds ratios (OR) and 95% confidence intervals (CI) were calculated using the major allele (A2) as a reference for all associations. The logistic regression analysis was adjusted for variables that were identified as potential covariates, namely: sex, BMI, fasting glucose levels and geographical origin of participants.

### Polygenic risk score (PRS)

A simple additive PRS for lower LDL-cholesterol was calculated using six variants (P<0.05) that were significant after adjusting for covariates (Fig 3A). A frequency plot with the PRS for cases and controls was generated. A t-test was completed to test for significance between cases and controls. A plot showing the linear correlation of the PRS against the mean of LDL-cholesterol level per risk score was generated (Fig 3B).

### Replication study

Replication analysis of the six variants associated with LDL-cholesterol was performed in the AADM study. This ongoing genetic epidemiology study of diabetes and related traits has been described previously [26,27]. Briefly, individuals attending medical clinics or referred for clinical suspicion of diabetes to university medical centres in urban sites in Nigeria (Enugu, Lagos, and Ibadan), Ghana (Accra and Kumasi), and Kenya (Eldoret) were recruited. Within the AADM study population, 50.2% were found to have Type 2 Diabetes. Genotyping was conducted using two different GWAS arrays: Affymetrix Axiom® PANAFR SNP array and the Illumina Consortium Multi-Ethnic Global Array (MEGA). Quality control was conducted separately for each of the resulting datasets. After technical quality control, sample-level genotype call rate was at least 0.95 for all participants. Each SNP dataset was filtered for missingness, HWE and allele frequency. SNPs passing the following filters were retained: missingness <0.05, HWE P>1 × 10^−6^ and MAF >0.01. SNPs that passed quality control were used as the basis for imputation. Imputation of all samples was done with the African Genome Resources Haplotype Reference Panel using the Sanger Imputation Server [39]. Analysis was conducted using a linear mixed model of the inverse normal transformations of the age-, age squared-, and sex-adjusted residuals. From prior work [40], the first three principal components (PCs) of the genotypes were found to be statistically significant and were included in the model, along with adjustment for BMI. The model included a genetic relationship matrix to account for the random effect of relatedness, as related individuals were included in AADM. Models were run using EPACTS [41]. Statistical significance was declared at P<0.01 (0.05/5 [variants available in AADM]) with consistent direction of effect.

### Statistics

A Chi-squared test was used to determine whether there was a significant difference between males and females with regard to LDL-cholesterol levels. The variables age, BMI, fasting glucose levels and LDL-cholesterol levels were all tested for normality. None of the variables fit a normal distribution, and therefore a Mann-Whitney U test was used to determine whether there was a significant difference between the cases and controls. STATA was used for these statistical tests [42].

## Results

There are more females in the low LDL-cholesterol group, and the high LDL-cholesterol group was characterised by a higher BMI and higher fasting glucose levels (Table 1).

**Table 1:** Phenotype characterisation of 1000 AWI-Gen participants and 4116 AADM participants.

	AWI-Gen			Replication cohort: AADM
Phenotype	High LDL-cholesterol (n = 500)	Low LDL-cholesterol (n = 500)	P value	(n = 4116)
**Sex (%F)**	49.40%	60.80%	3x10^-4^	59.8%
**Age (years) median (IQR)**	51(45.00–56.00)	50(45.00–55.00)	0.19	51.00(42.00–60.00)
**BMI (kg/m**^**2**^**) median (IQR)**	25.94(18.22–29.51)	20.73(19.04–23.27)	<1x	25.90(22.55–29.71)
**Fasting serum glucose (mmol/L) median (IQR)**	5.08(4.96–5.53)	4.60(4.19–5.05)	<1x10^-4^	7.31(4.61–8.56)
**LDL-cholesterol (mmol/L) median (IQR)**	4.21(3.93–4.61)	0.90(0.71–1.01)	NA	3.23(2.53–4.09)

IQR = Interquartile range

The distribution of the LDL-cholesterol values in the group with low LDL-cholesterol ranged from 0.4–1.2 mmol/L. The high LDL-cholesterol group had LDL-cholesterol levels ranging from 3.7–14.2 mmol/L. Two individuals were excluded from the analyses due to very high fasting LDL-cholesterol levels of 14.2 mmol/L and 8.23 mmol/L as they may have a monogenic FH aetiology.

### Variant filtering and QC

In total, 29 variants were selected for genotyping, but only 19 remained after assay design for final genotyping. Of these, five variants failed quality control parameters (four variants due to high missingness, one variant not in HWE), leaving 14 variants to be analysed (see Table 2 for more information). Seven samples were removed (five due to high missingness and two as they were high LDL-cholesterol outliers who could potentially have monogenic FH), leaving 993 samples to be analysed.

**Table 2:** Functional annotation scores and minor allele frequencies for 14 SNP variants.

**Low Density Lipoprotein Receptor (*LDLR*)**							
**Genomic location**	**rs number**	**PolyPhen2 score**	**SIFT score**	**CADD score**	**African MAF**	**Global MAF**	**Type of variant**
19:11238239	rs2569540	Probably damaging (0.96)	Deleterious (0)	1.20	C = 0.42	C = 0.32	missense
19:11242133	rs3826810	-	-	4.198	A = 0.12	A = 0.08	missense
19:11210921	rs72658855	-	-	15.14	T = 0.04	T = 0.01	synonymous
19:11226800	rs5929	-	-	12.1	T = 0.12	T = 0.12	synonymous
19:11230881	rs5925	-	-	0.51	C = 0.15	C = 0.34	synonymous
**Apolipoprotein B (*APOB*)**							
**Genomic location**	**rs number**	**PolyPhen2 score**	**SIFT score**	**CADD score**	**African MAF**	**Global MAF**	**Type of variant**
2:21229860	rs12720855	possibly damaging (0.64)	-	23.60	G = 0.08	G = 0.02	missense
2:21250914	rs679899	possibly damaging (0.64)	Tolerated (0.12)	26.60	A = 0.13	A = 0.49	missense
2:21260934	rs6752026	probably damaging (0.92)	Deleterious (0)	25.30	A = 0.11	A = 0.03	missense
2:21245367	rs3791981	-	-	2.20	G = 0.43	G = 0.20	regulatory region
**Proprotein convertase subtilisin/kexin type 9 (*PCSK9*)**							
**Genomic location**	**rs number**	**PolyPhen2 score**	**SIFT score**	**CADD score**	**African MAF**	**Global MAF**	**Type of variant**
1:55518370	rs7552471	-	-	20.40	T = 0.08	T = 0.02	synonymous
1:55509872	rs4927193	-	-	3.89	C = 0.22	C = 0.15	downstream gene
1:55518622	rs45613943	-	-	3.55	C = 0.29	C = 0.12	regulatory region
**Low Density Lipoprotein Receptor Adaptor Protein 1 (*LDLRAP1*)**							
**Genomic location**	**rs number**	**PolyPhen2 score**	**SIFT score**	**CADD score**	**African MAF**	**Global MAF**	**Type of variant**
1:25889539	rs12071264	-	-	4.70	G = 0.14	G = 0.04	intronic
1:25893927	rs35910270	-	-	4.73	Del = 0.42	Del = 0.49	frameshift

MAF = minor allele frequency, Del = deletion,— = no result available

Some missense variants had no SIFT or PolyPhen2 scores in the databases we used, since they were not annotated at the time the search was performed.

### Association analysis

**Fig 1:**
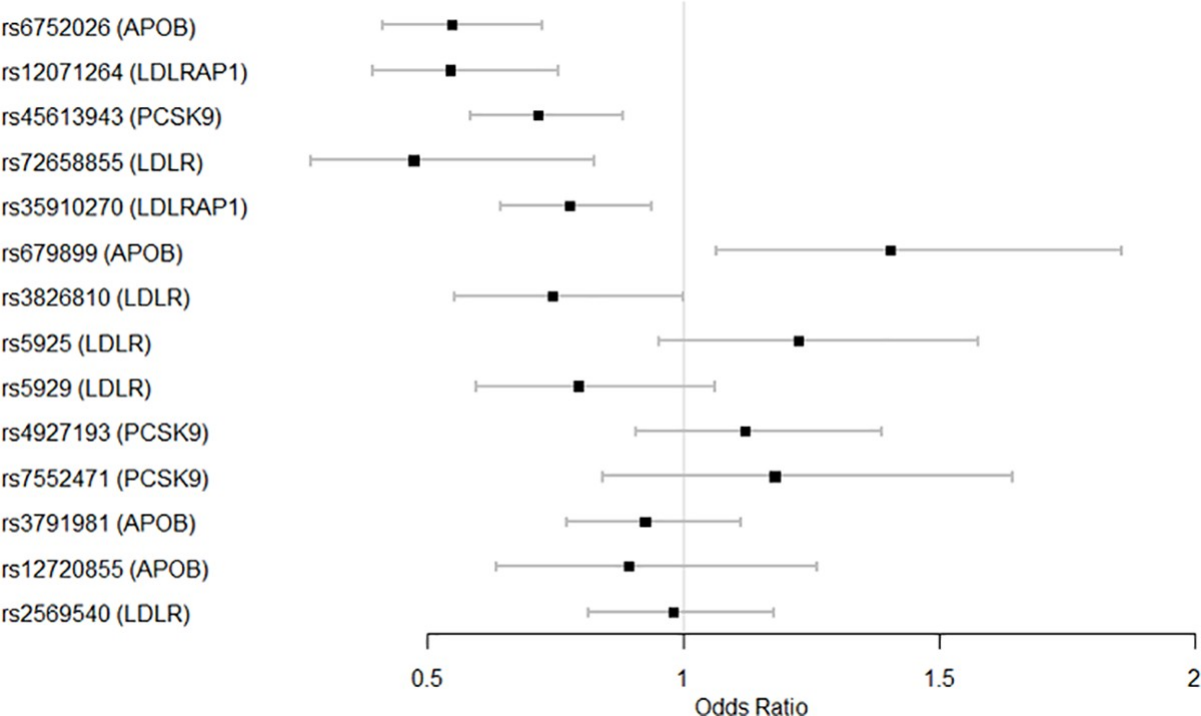
Logistic regression of the 14 selected gene variants associated with LDL-cholesterol levels. The plot shows the odds ratio (OR) for variants after adjusting for covariates (sex and geographic region). Bars represent 95% confidence interval (CI). When OR <1, the minor alleles are associated with low LDL-cholesterol levels. Only one variant is significantly associated with high LDL-cholesterol levels (rs679899) with a significant OR>1.

An allelic association (Table 3) found six significantly associated loci after correcting for multiple testing (P<0.05). The minor alleles of five variants were associated with low LDL-cholesterol levels and the minor allele of only one variant was associated with high LDL-cholesterol levels. After adjusting for covariates (sex, BMI, fasting glucose and geographic region), logistic regression (Table 4) revealed five variants that were significantly associated with low LDL-cholesterol levels: *APOB* rs6752026 (OR: 0.55) and *LDLRAP1* rs12071264 (OR: 0.54) and rs35910270 (OR: 0.78), *PCSK9* rs45613943 (OR: 0.72), *LDLR* rs72658855 (OR: 0.47). Only one variant was significantly associated with increased levels of LDL-cholesterol: *APOB* rs679899 (OR: 1.41). A forest plot was generated using the 14 variants from Table 4 (Fig 1).

**Table 3:** Allelic association of 14 variants with and without adjustment for multiple testing in 993 individuals.

Gene	SNP	Minor allele (A1)	Frequency in cases	Frequency in controls	OR	95% CI	Unadjusted P value	Adjusted P value
***APOB***	**rs6752026**	**A**	**0.10**	**0.17**	**0.56**	**0.43–0.73**	**1x10**^**-4**^	**2x10**^**-4**^
***PCSK9***	**rs45613943**	**C**	**0.24**	**0.32**	**0.67**	**0.55–0.82**	**1x10**^**-4**^	**5x10**^**-4**^
***LDLRAP1***	**rs12071264**	**G**	**0.07**	**0.11**	**0.56**	**0.40–0.76**	**2x10**^**-4**^	**0.001**
***APOB***	**rs679899**	**A**	**0.15**	**0.10**	**1.56**	**1.19–2.04**	**0.001**	**0.01**
***LDLR***	**rs72658855**	**T**	**0.02**	**0.04**	**0.48**	**0.28–0.82**	**0.01**	**0.02**
***LDLRAP1***	**rs35910270**	**del**	**0.37**	**0.43**	**0.80**	**0.67–0.96**	**0.01**	**0.04**
*LDLR*	rs3826810	A	0.09	0.12	0.73	0.55–0.97	0.03	0.06
*LDLR*	rs5929	T	0.10	0.13	0.75	0.57–0.99	0.04	0.07
*LDLR*	rs5925	C	0.16	0.14	1.20	0.94–1.54	0.15	0.23
*APOB*	rs3791981	G	0.46	0.49	0.90	0.75–1.07	0.24	0.34
*APOB*	rs12720855	G	0.07	0.08	0.87	0.62–1.21	0.41	0.52
*PCSK9*	rs7552471	T	0.09	0.08	1.08	0.79–1.48	0.64	0.75
*PCSK9*	rs4927193	C	0.24	0.24	1.02	0.83–1.26	0.84	0.85
*LDLR*	rs2569540	G	0.43	0.43	0.98	0.82–1.17	0.85	0.85

OR = odds ratio, 95% CI = 95% confidence interval

**Table 4:** Significant associations with LDL-cholesterol levels in the AWI-Gen cohort and replication in the AADM study.

			AWI-Gen participants					AADM participants			
Gene	Chr	SNP	A1	N	OR	95% CI	P value^1^	Frequency	Beta	SE	P value^2^
***APOB***	**2**	**rs6752026**	**A**	**993**	**0.5**	**0.41–0.73**	**2.82x10**^**-5**^	0.15	-0.06	0.032	0.08
***LDLRAP1***	**1**	**rs12071264**	**G**	**993**	**0.54**	**0.39–0.76**	**2.73x10**^**-4**^	0.10	0.02	0.039	0.67
***PCSK9***	**1**	**rs45613943**	**C**	**992**	**0.72**	**0.58–0.88**	**0.001**	**0.30**	**-0.10**	**0.025**	**<9x10**^**-5**^
***LDLR***	**19**	**rs72658855**	**T**	**993**	**0.47**	**0.27–0.82**	**0.008**	0.05	0.0019	0.053	0.97
***LDLRAP1***	**1**	**rs35910270**	**G**	**993**	**0.78**	**0.64–0.94**	**0.008**	**-**	**-**	**-**	**-**
***APOB***	**2**	**rs679899**	**A**	**992**	**1.41**	**1.06–1.86**	**0.016**	0.11	0.06	0.036	0.092

A1 = minor allele, N = no. of individuals genotyped, OR = odds ratio, 95% CI = 95% confidence interval, SE = standard error,

^1^ Adjusted for multiple testing and covariates (sex, BMI, fasting glucose levels and geographic region). Statistical significance set at P<0.05,

^2^ Adjusted for covariates (three PCs, BMI and relationship matrix). Statistical significance set at P<0.01 (P<0.05/number of variants tested).

**Fig 2:**
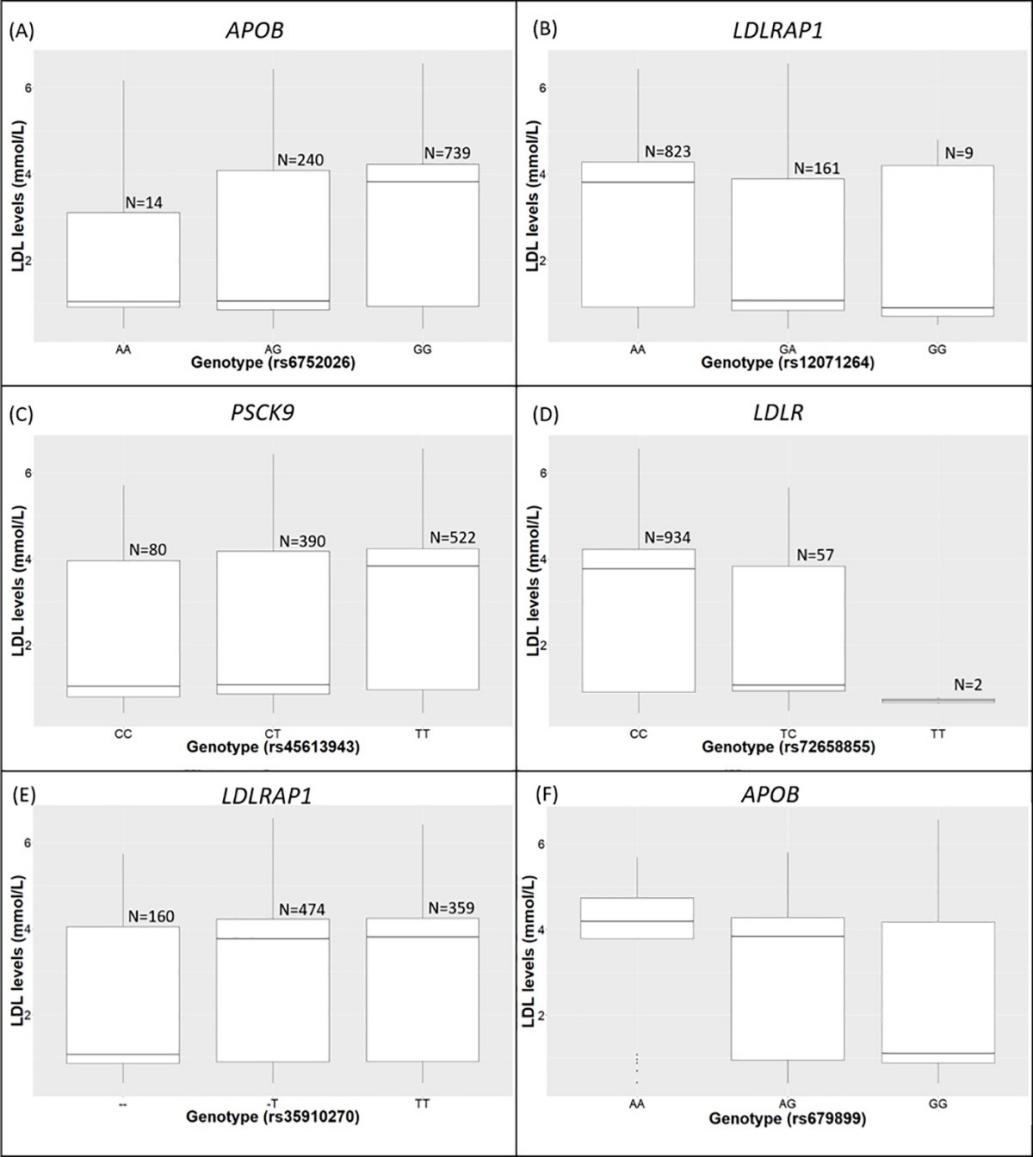
Genotypes relative to LDL-cholesterol distribution for 6 variants significantly associated with LDL-cholesterol levels after logistic regression. The 993 AWI-Gen individuals with low and high LDL-cholesterol are included in the plots. **A-D:** *APOB* rs6752026, *LDLRAP1* rs12071264, *PCSK9* rs45613943 and *LDLR* rs72658855 show how LDL-cholesterol levels decrease with presence of the minor allele. **E:** *LDLRAP1* shows how LDL-cholesterol levels decrease only when both minor alleles are present (deletion). **F:** *APOB* rs679899 shows an increase of LDL-cholesterol levels with the presence of the minor allele.

Fig 2 shows the association of the genotypes for six variants significantly associated with low and high LDL-cholesterol levels after adjusting for covariates. For four variants (Fig 2A–2D), the minor allele contributes to lower LDL-cholesterol levels in these populations. There is a decrease in LDL-cholesterol when the minor allele is present (in both the heterozygous and homozygous genotype) for these four variants. This suggests that these alleles may have a gain of function LDL-cholesterol lowering mode of action. The minor allele of the fifth variant (rs35910270) (Fig 2E) shows that there is a decrease in LDL-cholesterol levels only when the homozygous minor allele genotype is present. This suggests a loss of function, recessive mode of action. The major allele is associated with high LDL-cholesterol levels for the five variants. The minor allele of the final variant (rs679899) (Fig 2F) shows that the minor allele is associated with high LDL-cholesterol levels.

**Fig 3:**
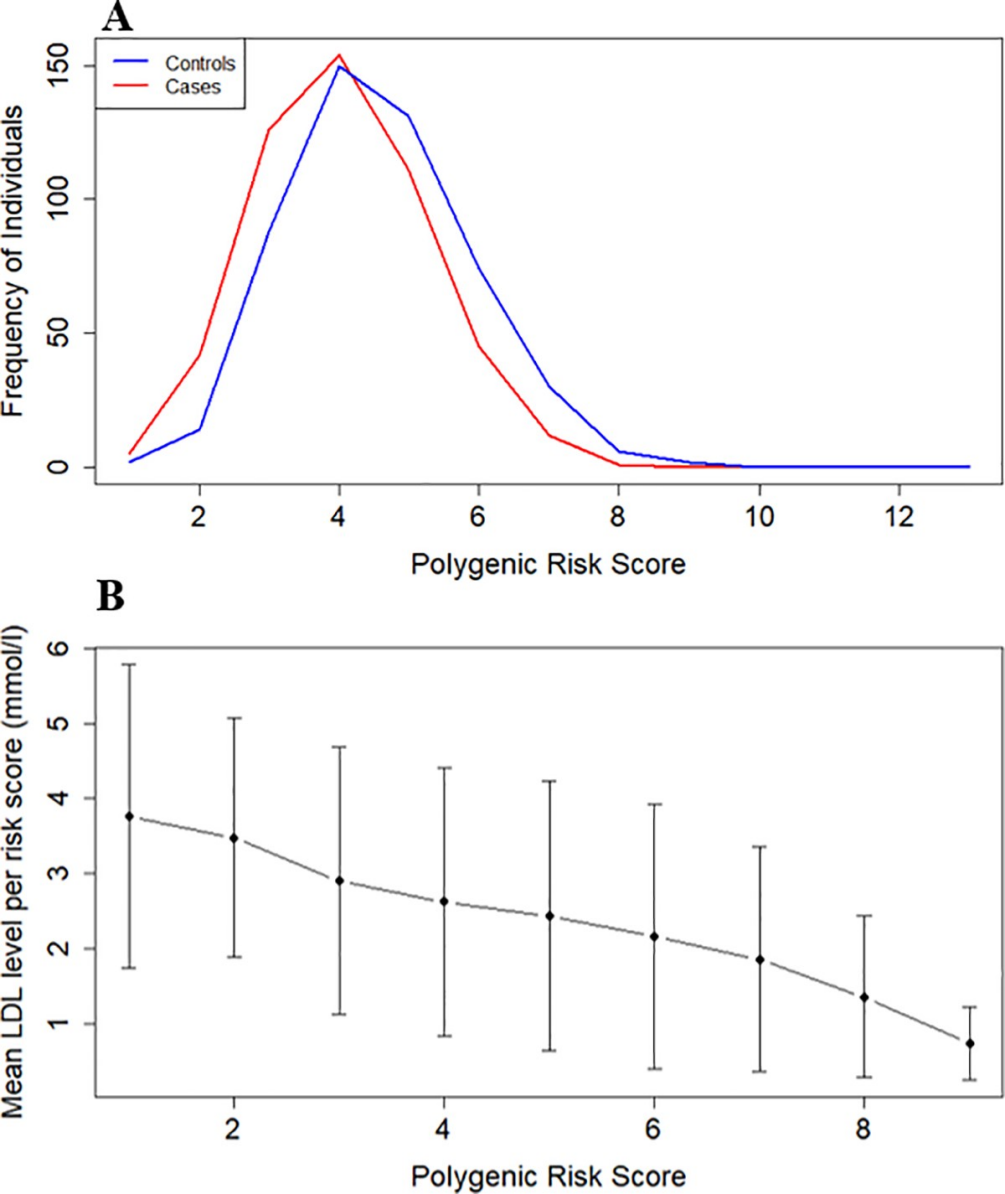
Correlation of the polygenic risk score (PRS) with LDL-cholesterol levels in 993 individuals. Six LDL-cholesterol associated variants (P<0.05) after adjustment for covariates. Cases include individuals with high LDL-cholesterol levels, and controls with low LDL-cholesterol levels. **A:** PRS calculated using six variants. Risk score refers to the number of lipid lowering alleles. Plot shows the frequency of cases and controls for each score. The curve of the controls is shifted to the right, indicating that in controls the LDL-cholesterol levels decreases with the addition of alleles associated with lower LDL-cholesterol levels (either common or minor allele). **B:** Plot of risk score against mean LDL-cholesterol level per risk score. It is apparent that with the addition of each allele associated with lower LDL-cholesterol levels (common or minor allele), the mean LDL-cholesterol level of the participants decreased.

In the PRS, “risk” is depicted by lower LDL-cholesterol (Fig 3A). Therefore, the curve of the controls (low LDL-cholesterol) is shifted to the right (higher risk score for low LDL-cholesterol), as expected. The two groups are significantly different from each other (P = 0.001). Fig 3B shows the correlation of the PRS with LDL-cholesterol levels. It is apparent that individuals with a greater number of LDL-cholesterol reducing alleles have lower LDL-cholesterol levels. Alleles individually have a small effect on the phenotype, but when considering alleles across all five loci, the additive effect is clearly observed.

### Replication of associated variants in an independent African study

We evaluated our associated variants in an independent sample of West and East Africans drawn from the AADM study (participant characteristics: Table 1). One of the variants, rs35910270 (*LDLRAP1*), did not pass quality control filters in the replication dataset and was not included in the replication analysis. *PCSK9* variant rs45613943 was associated with lower LDL-cholesterol in the AADM data (P<9x10^-5^; Table 4). The association of both *APOB* variants was directionally consistent with the main findings but did not reach statistical significance (rs6752026 P = 0.08; rs679899 P = 0.09; statistical significance set at P<0.01). There was no association between rs12071264 or rs72658855 with LDL-cholesterol in the AADM study.

## Discussion

The aim of this study was to examine potentially functional variants in four genes for association with LDL-cholesterol levels in black African populations. To increase the power to detect associations we selected participants at the extremes of the LDL-cholesterol distribution with high and low levels. LDL-cholesterol levels are influenced by many genetic variants at different loci and by environmental factors, and lipid levels have an estimated heritability ranging between 40 and 60% [43]. GWAS studies of very large sample sizes have generally explained only 10–12% of the variability in LDL-cholesterol levels [18]. Some of the missing heritability could be explained by gene-environment interactions and gene-gene interactions [44].

Mutations in the genes investigated also contribute to monogenic dyslipidaemias. Deleterious mutations in *LDLR* are the most common cause of FH [8,45,46]; loss of function variants in *LDLRAP1* have been documented to cause high LDL-cholesterol levels with a recessive form of inheritance (38); variants in *APOB* have been known to cause both low and high LDL-cholesterol levels [16,47–49]; and loss of function variants that cause low LDL-cholesterol have been identified in *PCSK9* [50–52]. In this study, two *LDLRAP1* variants were associated with low LDL-cholesterol levels. *LDLRAP1* rs12071264 is located in intron 5, close to a splice site [53], and could affect transcription. This variant is absent in European populations. *LDLRAP1* rs35910270 is in the 3’UTR and is common in both European (47%) and African (42%) populations.

Two variants in *APOB* were associated with LDL-cholesterol levels. rs6752026, a missense variant in exon 5, was associated with lower LDL-cholesterol levels. The proline to serine change is predicted to be deleterious by both SIFT and PolyPhen, however, it has not been previously associated with LDL-cholesterol levels. This variant occurs at very low frequencies in European populations (~0.1%) but is common in Africans (~11%). The second, rs679899, is an alanine to valine missense variant associated with higher LDL-cholesterol levels. It is common in European populations (86%), but less common in African populations (13%). The evidence for the deleterious nature of the variant is conflicting with PolyPhen predicting it to be possibly damaging, while SIFT predicts it to be tolerated. One variant in *PCSK9*, rs45613943, was associated with low LDL-cholesterol levels and was also significantly associated with low LDL-cholesterol levels in an African replication cohort (Beta:-0.10, P<9x10^-5^), strengthening its association with low LDL-cholesterol levels across several African populations. The variant allele occurs at low frequencies in European populations (5%) but is more common in Africa (~29%). This is likely to be a loss of function variant, as loss of function of the PCSK9 enzyme increases the number of LDL receptors returning to the surface of the cell, but further functional studies would be required to assess the effect of the variant on the function of the protein. Interestingly, one variant in *LDLR*, rs72568855, was associated with low LDL-cholesterol levels and this variant has not been reported in European populations.

Associations with four of the variants would not have been detected in studies with participants from Europe as they appear to be African-specific or extremely rare in Europeans. In all cases, the rare allele was associated with lower LDL-cholesterol. This may suggest that the allelic variants, excluding rs45613943, have a gain of function impact, or are in close LD with functional variants that contribute to decreased LDL-cholesterol levels in Africans. The *PCSK9* variant, rs45613943, is a regulatory variant. This may decrease transcription, resulting in less production of protein and an increased turnover of the LDL receptors, thereby reducing the serum levels of LDL-cholesterol. The *APOB* rs679899 rare allele was the only variant that was significantly associated with high LDL-cholesterol levels in this study.

Participants from East, West and South Africa have an LDL-cholesterol distribution favouring lower LDL-cholesterol levels and interestingly, the rare alleles at five loci (Fig 2A–2E) showed association with low LDL-cholesterol levels. Only one variant was associated with high LDL-cholesterol levels (Fig 2F). The PRS shows a modest, but significant (P = 0.001) shift between individuals with high and low LDL-cholesterol levels and a PRS is likely to improve with more markers from a GWAS analysis of the full AWI-Gen cohort.

The LDL-cholesterol distribution in African populations is generally considered to be lower, compared to non-African populations; therefore, it is counter intuitive that the common alleles at the five associated variants would associate with higher LDL-cholesterol levels in Africans. For five of the six significantly associated variants identified in this study, the major alleles were associated with higher LDL-cholesterol levels. Although this may suggest that the normal distribution of LDL-cholesterol levels in African populations would be expected to be higher, the rare alleles in rs6752026, rs12071264, rs35910270 and rs72658855 may have some gain of function effect that associates them with lower LDL-cholesterol levels.

In addition, gene-environment interactions could play a role, and low-fat diets and high physical activity could also contribute to lower LDL-cholesterol levels in African populations. However, as African populations become more urbanised, a more western lifestyle will follow, which could increase LDL-cholesterol, especially in those with a genetic predisposition for high LDL-cholesterol levels [54].

Detecting hyperlipidaemia early in individuals and administering treatment and lifestyle changes can reduce the number of CVD related events, and subsequently reduce the health burden among Africans [55]. Precision public health is using data to implement intervention strategies that will most efficiently benefit the majority of individuals in a population [56]. Using population specific genetic variants to predict LDL-cholesterol levels will only be effective if they have good predictive potential and the assays are affordable. At present, a serum cholesterol test remains a better and more cost-effective measure of LDL-cholesterol levels. Intervention strategies, such as lifestyle changes and appropriate prescription of medication for high LDL-cholesterol that is effective for the population in question, could be implemented for a better outcome.

### Limitations & future research

Even though the AWI-Gen participants were all African, they were multi-ethnic with an uneven distribution across geographic regions in West, East and South Africa. This could have caused a bias due to population sub-structure, despite adjusting for study site (as proxy for ethnicity) in the logistic regression analysis. Nonetheless, since lipid data on African populations are limited, this study serves as a starting point for subsequent research endeavours on understanding genetic associations with LDL-cholesterol levels in African populations.

Due to funding limitations, only a small number of variants were tested per gene. Ideally, a more representative set of markers to capture all the haplotype blocks across each gene (to account for lower linkage disequilibrium in African populations) would have provided a more accurate indication of the association of variants in these genes with LDL-cholesterol levels. A GWAS analysis for LDL-cholesterol in the AWI-Gen study is in progress.

This study used a basic logistic regression approach to analysing variants, unlike the linear mixed model used by the replication study. Glucose levels were not adjusted for in the replication study.

## Conclusion

In selected African populations in four sub-Saharan African countries, we investigated the association of variants in four genes (*LDLR*, *APOB*, *PCSK9* and *LDLRAP1*) known to be involved in lipid metabolism. The study identified five variants associated with low LDL-cholesterol levels and one variant associated with high LDL-cholesterol levels. Using a different cohort from West Africa, we replicated the association of *PCSK9* rs45613943C with low LDL-cholesterol. These data suggest allelic association differences with LDL-cholesterol levels across African populations, which may be influenced by gene-environment interactions.

## Supporting information

S1 File (MAP)

S2 File (PED)

S3 File (TXT)
