# Correction: Genetic associations between serum low LDL-cholesterol levels and variants in *LDLR*, *APOB*, *PCSK9* and *LDLRAP1* in African populations

**DOI:** 10.1371/journal.pone.0249478

**Published:** 2021-03-26

**Authors:** Mahtaab Hayat, Robyn Kerr, Amy R. Bentley, Charles N. Rotimi, Frederick J. Raal, Michèle Ramsay

There is an error in Table 1. The P value for BMI is incorrect. The P value for BMI should be <1x10^-4^.

**Table 1:** Phenotype characterisation of 1000 AWI-Gen participants and 4116 AADM participants.

	AWI-Gen			Replication cohort: AADM
Phenotype	High LDL-cholesterol (n = 500)	Low LDL-cholesterol (n = 500)	P value	(n = 4116)
**Sex (%F)**	49.40%	60.80%	3x10^-4^	59.8%
**Age (years)** **median (IQR)**	51(45.00–56.00)	50(45.00–55.00)	0.19	51.00(42.00–60.00)
**BMI (kg/m**^**2**^**)** **median (IQR)**	25.94(18.22–29.51)	20.73(19.04–23.27)	<1x10^-4^	25.90(22.55–29.71)
**Fasting serum glucose (mmol/L)** **median (IQR)**	5.08(4.96–5.53)	4.60(4.19–5.05)	<1x10^-4^	7.31(4.61–8.56)
**LDL-cholesterol (mmol/L)** **median (IQR)**	4.21(3.93–4.61)	0.90(0.71–1.01)	NA	3.23(2.53–4.09)

IQR = Interquartile range
